# Enhancing Knowledge Sharing Management Using BIM Technology in Construction

**DOI:** 10.1155/2013/170498

**Published:** 2013-11-07

**Authors:** Shih-Ping Ho, Hui-Ping Tserng, Shu-Hui Jan

**Affiliations:** Department of Civil Engineering, National Taiwan University, Number 1, Setion 4, Taipei 10617, Taiwan

## Abstract

Construction knowledge can be communicated and reused among project managers and jobsite engineers to alleviate problems on a construction jobsite and reduce the time and cost of solving problems related to constructability. This paper proposes a new methodology for the sharing of construction knowledge by using Building Information Modeling (BIM) technology. The main characteristics of BIM include illustrating 3D CAD-based presentations and keeping information in a digital format and facilitation of easy updating and transfer of information in the BIM environment. Using the BIM technology, project managers and engineers can gain knowledge related to BIM and obtain feedback provided by jobsite engineers for future reference. This study addresses the application of knowledge sharing management using BIM technology and proposes a BIM-based Knowledge Sharing Management (BIMKSM) system for project managers and engineers. The BIMKSM system is then applied in a selected case study of a construction project in Taiwan to demonstrate the effectiveness of sharing knowledge in the BIM environment. The results demonstrate that the BIMKSM system can be used as a visual BIM-based knowledge sharing management platform by utilizing the BIM technology.

## 1. Introduction

It is vitally important for project managers and jobsite engineers to obtain knowledge about construction and to solve any problems that may arise. To achieve this knowledge, jobsite engineers can learn from the experience of other jobsite engineers. Construction experience transfer involves using knowledge gained during the completion of previous projects to maximize the achievement of current project objectives [1]. In order to share knowledge between similar projects, construction professionals have traditionally used techniques ranging from annual meetings to face-to-face interviews [1]. In addition to experts' memory, construction experience can be recorded in various media, such as documents, databases, and intranets. Knowledge management (KM) is the collection of processes controlling the creation, storage, reuse, evaluation, and use of experience-based knowledge in a particular situation or problem-solving context. In construction, KM focuses on the acquisition and management of important experience-based knowledge provided by job engineers.

Regardless of whether a project executed by an architectural firm is successful, valuable knowledge can be gained and should be documented so that jobsite engineers can identify what worked and what did not. From the perspective of KM in construction, these experiences and the knowledge gained from them are valuable, as they are accumulated through large investments in manpower, time, and money. Most jobsite engineers agree that KM in construction projects is a vital tool construction management. The sharing of knowledge and feedback provided by jobsite engineers help to prevent mistakes that have been made in previous projects. Drawing on knowledge and experience thus eliminates the need to solve many problems from scratch.

Most recent construction projects in Taiwan have applied KM systems to improve construction management during the construction phase. However, most of the shared information during the construction phase is in the form of text-based information, with less focus on virtual illustration and sharing. In construction projects, KM may involve many important relationships between the presentation and retrieval of knowledge and CAD. Furthermore, when knowledge is available for sharing, it is not easy for engineers to understand it directly without 2D or 3D CAD illustrations. Building Information Modeling (BIM) is the process of generating and managing data during the building life cycle [2]. BIM technology has the potential to enable fundamental changes in project delivery that will support a more integrated, efficient process [3]. BIM digitally contains precise geometry and relevant data needed to support the design, procurement, fabrication, and construction activities used to build 3D object-oriented CAD [4]. The main characteristics of BIM include illustrating 3D CAD-based presentations, keeping information in digital format, and facilitating the easy updating and transfer of information in a BIM environment.

**Figure 1:**
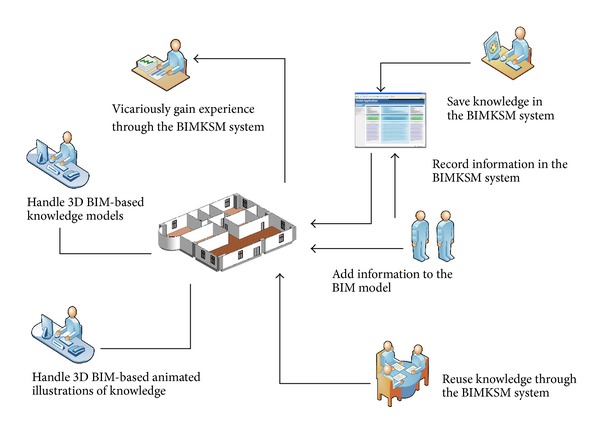
The application of the visual knowledge management in construction.

The primary purpose of the study is to develop a way for jobsite engineers to effectively acquire, manage, and reuse knowledge gained from other jobsite engineers and integrated using the BIM technology within the 3D CAD environment. The study proposes a novel approach using BIM-based knowledge models integrated with the BIM technology to track and manage valuable experience-based knowledge. The main function of the BIM technology in this study is the 3D BIM-based illustration of experience-based knowledge. The proposed BIM-based animated illustration of knowledge is applied to keep and explain information in a digital format and to facilitate the updating and transfer of knowledge in the BIM environment. Using BIM-based animated illustrations of information, engineers can get an overview of previous scenarios in selected projects and use the knowledge gained for future construction work and understand the setup/process from their own professional perspective. Project managers and jobsite engineers can track and access the most recently shared information on relevant issues during the construction phase. Knowledge of various issues can be updated quickly and made available to each participant in the visual environment. This study addresses the application of knowledge sharing management and proposes a BIM-based Knowledge Sharing Management (BIMKSM) system for project managers and engineers. The research is a pilot study that involves applying the BIMKSM system for knowledge management to a building project in Taiwan (see Figure 1), in order to analyze and discuss the whole process of construction knowledge sharing management and to implement the KM sharing during the construction phase. The processing and content of construction sharing experience-based knowledge can be modified according to a project's particular characteristics. Finally, the BIMKSM system is then applied in a selected case study to verify the effectiveness of sharing knowledge in the BIM environment.

In sum, the purpose of this study is to (1) help jobsite engineers collect and share knowledge effectively through the BIMKSM system during the construction phase; (2) help engineers to refer to and exchange knowledge from the jobsite engineers using the BIMKSM system during the construction phase; (3) develop a web-based KM system to improve the exchange and tracking of knowledge for project managers and jobsite engineers.

## 2. Background Research 

### 2.1. Knowledge Management in Construction

Knowledge management deals with collecting, modeling, storing, reusing, evaluating, and maintaining knowledge [5–7]. Numerous research efforts have focused on applications of knowledge management in construction. El-Diraby and Kashif [8] presented a distributed ontology architectural design developed by rigorous knowledge acquisition and ontology development techniques for KM in the highway construction industry. Hartmann and Fischer [9] described how project teams can use 3D/4D models efficiently to support the communication of knowledge during the constructability review on construction projects. Ribeiro [10] analyzed KM effort based on case studies and provided recommendations and insights for enhancing KM in construction firms. Chen and Mohamed [11] provided empirical evidence for the stronger strategic role of tacit KM in comparison to explicit KM. Kivrak et al. [12] used a survey to find out how tacit and explicit knowledge are captured, stored, shared, and used in forthcoming projects and to identify major drivers and barriers in knowledge management. Chen et al. [13] presented a knowledge-sharing model to determine whether risk mitigation based on the use of derivatives would be beneficial to the companies. Forcada et al. [14] presented a survey of perceptions of KM implementation in the Spanish construction sector and compares the results obtained from design and construction firms.

### 2.2. BIM Application in Construction

A great deal of previous research pertains to BIM issues in construction. Tse et al. [15] discussed the core barriers and recommended using BIM technology for construction industries. Mah et al. [16] proposed rapid computations of CO_2_ emissions from various house sizes, designs, and materials integrated with BIM technology. Goedert and Meadati [17] extended BIM technology into the construction process to create a single repository of facility data for the owner. Succar [18] explored publicly available international guidelines and introduced the BIM framework as a research and delivery foundation for industry stakeholders. Dossick and Neff [19] examined the use of BIM technologies for mechanical, electrical, plumbing, and life safety systems. Ren et al. [20] proposed a framework for integrating BIM for quantity takeoff and cost-estimating applications with e-commerce solutions for material procurement and supplier performance evaluation. Davies and Harty [21] developed BIM-enabled tools to allow site workers using mobile tablet personal computers to access design information and to capture work quality and progress data on-site. Martins and Monteiro [22] developed BIM-based automated code-checking procedures and system for water distribution systems. Zhang et al. [23] developed automated safety checking platform integrated BIM that informs construction engineers and managers for preventing fall-related accidents before construction starts. Wong et al. [24] highlighted critical initiatives derived from the review of BIM implementations in both the public and private sectors in six selected countries. Bryde et al. [25] explored the extent to which the use of BIM has resulted in reported benefits on a cross-section of construction projects. Succar et al. [26] proposed the five BIM framework components for the design, construction, and operation (DCO) stakeholders to measure and improve their BIM performance. Sebastian and Van Berlo [27] generated an instrument for benchmarking BIM performance to provide insight into the current BIM performance level of design, engineering, and construction firms. Bynum et al. [28] investigated the perceptions of the use of BIM for sustainable design and construction among designers and constructors. Wang et al. [29] explored how BIM will beneficially support facility management in the design phase, such as space planning and energy analysis. Zhang et al. [30] proposed and verified Industrial Foundation Classes-based graphic information model as the foundation of data sharing in virtual construction systems and in other AEC/FM applications.

Although numerous knowledge management systems have been developed for the application of construction knowledge management, such systems typically exist for knowledge sharing using only text-based illustrations. To enhance construction-related knowledge sharing using a BIM-based environment, this study proposes a novel management system for project managers and jobsite engineers.

## 3. Methodology 

### 3.1. BIM-Based Knowledge Management Approach

In this study, the proposed BIMKSM system facilitates visual knowledge sharing and management using the BIM technology during the construction phase. The BIM technology stores any problems, solutions, and comments, allowing project managers and jobsite engineers to access the most up-to-date knowledge. The primary advantages of the proposed BIMKSM system are (1) to effectively link knowledge using BIM-based graphic representations; (2) to promote relationships between areas of expertise via both vertical and horizontal graphic representations; and (3) to provide statuses of acquired knowledge of different situations using different colors.

Most knowledge can be classified as either tacit or explicit knowledge. Tacit knowledge is personal, context-specific knowledge that is difficult to formalize, record, or articulate. This type of knowledge is stored in the heads of people [31]. Tacit knowledge or experience is primarily developed through a process of trial and error in practice. Tacit knowledge that can be communicated directly and effectively is personal knowledge embedded in individual experience and shared and exchanged through direct, face-to-face contact [32]. In contrast, the acquisition of explicit knowledge is indirect; it must be decoded and recoded into one's mental models and is then internalized as tacit knowledge. Explicit knowledge can be codified and transmitted in a systematic and formal language. Explicit knowledge can be found in the documents of organizations, including reports, articles, manuals, patents, pictures, images, video, audio, software, and other forms. In this study, “tacit knowledge” refers to “hard” information that is visibly or invisibly related to part of an area of knowledge, including experience and know-how. Explicit knowledge is “soft” information that enables or facilitates the execution of specific information, including contracting, drawing, solving problems, or approving proposals. All jobsite engineers are responsible for sharing knowledge pertaining to their own domain. Any BIM model whose integrated information/knowledge sharing requirements have been noted will be classified as explicit in order to allow relevant experiences and processes to be recorded. Therefore, the shared information associated with objects of BIM model can be referred to and reused in other projects.

Shared information from all jobsite engineers is divided and saved as “activity,” “object,” or “issue” for collection and management. The main advantage of BIM-based knowledge management is the ease with which information and knowledge can be understood and reapplied. Knowledge saved in the “issue” category includes both tacit and explicit knowledge. With respect to explicit knowledge, BIM-related information normally includes original comments, reports, drawings, documents, and comments submitted by jobsite engineers. In contrast, tacit knowledge may include process records, problems faced, problems solved, expert suggestions, know-how, innovations, and notes on experience. Such information is better saved in issue-based components to facilitate classification and searching by users. Information that relates to the whole project that cannot be easily classified into issue components is saved under the “project” category.

**Figure 2:**
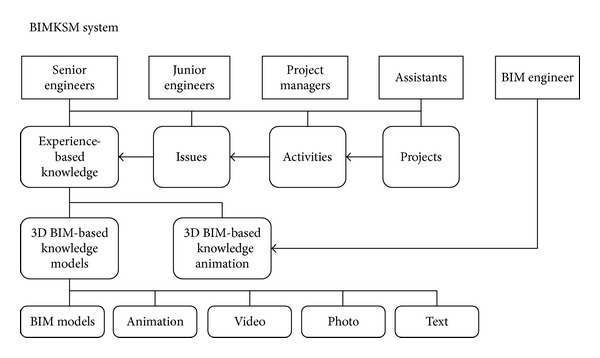
The concept and framework of the BIM-based knowledge models.

A BIM-based knowledge model can be defined as a graphic representation of experiences linking relationships between objects of the BIM model and aspects of experience-based knowledge. The BIM technology retains knowledge in a digital format, facilitating easy updating and transfer of knowledge into the BIM environment. A BIM-based knowledge model is designed to be easily integrated with experience-based information and objects of the model (see Figure 2). Information in the BIM-based knowledge models can be identified, tracked, and managed, and problems encountered during construction projects can be solved. The most up-to-date knowledge and solutions can be acquired from participating engineers and then shared and saved as objects of the BIM model for future reference. The model is constructed from variables that can be decomposed into objects of a BIM model and can then store the identified knowledge. Information stored in the objects of BIM models includes both facing problems and solutions. Facing problems may be knowledge issues, knowledge attributes, descriptions of problems, or knowledge attachments (e.g., documents, reports, drawings, and photographs).

### 3.2. Procedures of BIM-Based Knowledge Models Usage

**Figure 3:**
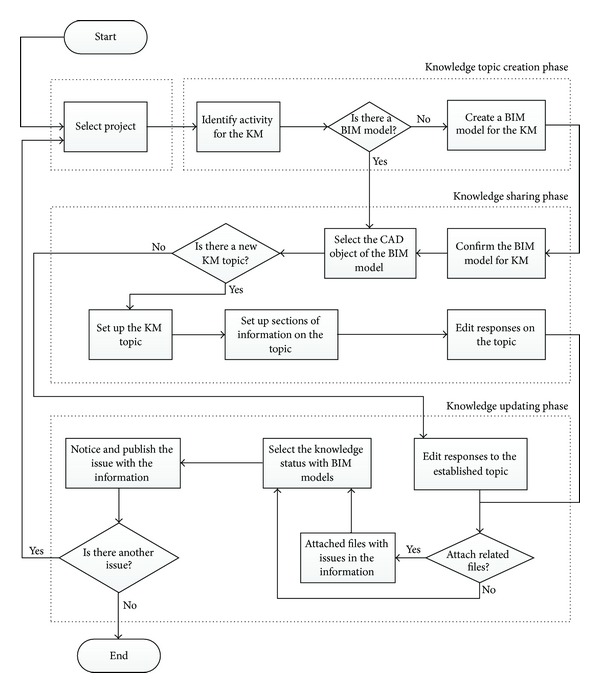
A flowchart of the procedure of the knowledge models usage.

The procedures for using BIM-based knowledge models are based on a knowledge management framework. The procedure consists of three phases: creating an issue, sharing knowledge, and updating said knowledge.  Figure 3 presents the flowchart of the procedure of the knowledge models usage for knowledge sharing.

#### 3.2.1. Issue Creation Phase

The initial engineer may determine which projects, activities, and issues are suitable for knowledge sharing. Furthermore, the issue must be set up by the initial participant (engineer) at the beginning of the phase. Such information under knowledge issues includes determining the type of knowledge, objects of BIM model, activities, and projects that should be assigned in association with the issue.

#### 3.2.2. Knowledge Sharing Phase

After studying the published materials, all qualified and interested engineers are invited to edit and submit any knowledgeable comments they may have on the issue. All explicit knowledge prepared by engineers needs to be digitized by them or by assistants before it can be submitted to the BIMKSM system. All knowledge must also be examined and confirmed before publishing. All interested engineers can discuss problems related to selected issues and objects of the BIM model and seek responses from other engineers and managers through the BIMKSM system. Meanwhile, the engineers can direct responses either to individuals or a group. After tacit and explicit knowledge is saved in the system, all knowledge can be referenced and reused. Engineers can gain knowledge from the issues catalogue of the objects and can access this catalogue for use in other similar projects.

#### 3.2.3. Knowledge Updating Phase

After applying tacit and explicit knowledge to similar projects, the engineers can resolve their problems related to those issues. Finally, the engineers can note and submit the new tacit knowledge and record the experiences through which it was gained, and they can associate this information with the original knowledge. Furthermore, the information is updated again because further feedback and updated knowledge are provided regarding the issues. After the approval process has been completed, the updated knowledge set is republished to authorized members.

## 4. System Implementation 

### 4.1. System Architecture

The BIMKSM system provides a user friendly portal for all project participants. It also serves as a real-time online communication channel for knowledge management. All data are stored and classified using BIM-based knowledge models. The BIMKSM system is a solution that uses a single unified database linked to BIM files with different levels of access granted to users based on their roles. Authorized participants can access the BIM-based knowledge models to update information on content relevant to the user's responsibilities in the project. When information is updated in the BIMKSM system, the server automatically sends e-mails to the project managers and jobsite engineers involved in the project.

The developed BIMKSM system runs on Microsoft Windows 2008 software with an Internet Information Server (IIS) as the web server. The BIMKSM system was developed using Java Server Pages (JSPs), which are easily incorporated with HTML and JavaScript technologies. The BIMKSM system server supports four distinct layers: interface, access, application, and database layers. Each layer has its own responsibilities. The interface layer defines administrative and end-user interfaces. Users can access information via web browsers such as Microsoft Internet Explorer or Google Chrome. Administrators control and manage information via the web browser or a separate server interface. The access layer provides system security, restricted access, firewall services, and system administration functions. The application layer defines various applications for analyzing and managing information. The database layer consists of a primary Microsoft SQL Server 2003 database. A firewall and virus scan can be used to protect the system database against intrusion. Users can utilize the BIM models in the BIMKSM system to request assistance or send word of a problem directly to the BIMKSM system to ask for further support.

**Figure 4:**
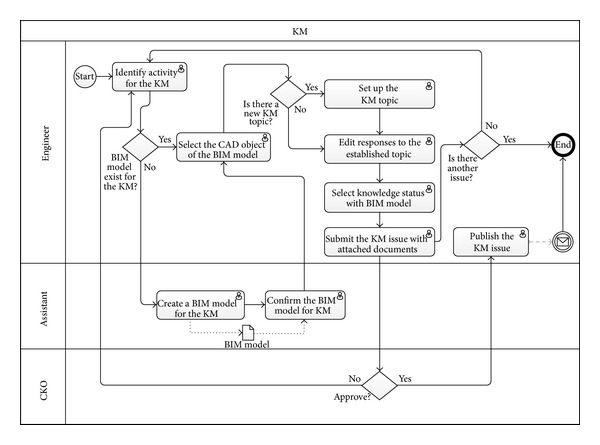
The BIM-based knowledge sharing and management process flowchart.

In this study, the BIM model is interpreted as an information model in the BIMKSM system. The application of BIM models to acquire and store information about an object involve a description of the problem being faced, knowledge, comments, and attaching documents in the BIMKSM system. Autodesk Revit Architecture and Revit MEP were used to create the BIM model and files. Autodesk Design Review was used to read the files for BIM-based knowledge models. Also, Autodesk Inventor was used to create BIM-based animations to illustrate the knowledge. The integration of the information with the BIM models was achieved using the Autodesk Revit application programming interface (API) and the Microsoft Visual Basic. Net (VB.Net) programming language. The BIM-based knowledge models were developed in Autodesk Revit Architecture and Revit MEP by programming in VB.Net and using Revit API. All information in the BIM files could be exported to an ODBC database for connection with the BIMKSM system.  Figure 4 shows the BIM-based knowledge sharing and management process flowchart in the BIMKSM system.

### 4.2. System Modules

All modules in the system are briefly described below.

#### 4.2.1. Authority Setup Module

The authority setup module is an access control mechanism preventing unauthorized users from entering the system or retrieving sensitive information. The BIMKSM system requires all project participants (managers and jobsite engineers) to register by providing a unique user ID and password for authentication.

#### 4.2.2. Knowledge Edition Module

Through this module, project participants can edit their relevant contributions to the objects in the BIM model. Generally, participants may create a new issue or contribute to those started by others in order to share their knowledge on various aspects of the project. The edited information will be saved in issues by categories associated with the relevant objects of the BIM model. Also, attached documents and report files must be uploaded in PDF format, the standard file format. The knowledge edition module allows experts and engineers to share issue-based tacit knowledge via a discussion forum.

#### 4.2.3. Alert Setup Module

This module helps project participants set up an alert service for monitoring knowledge via e-mail. Dates related to any notification of new information are submitted or updated systematically and project participants can determine who is invited to submit knowledge.

#### 4.2.4. Report Module

The report module allows users to easily access the summarized information to identify needs and analyze what has been recorded. The knowledge report can be illustrated with a BIM model, a description, and a summary of the information. Furthermore, all reports can be presented on the web or extracted to PDF format. This process allows users to make and organize knowledge-related reports from a central location.

## 5. Case Study 

### 5.1. Case Study Introduction

**Figure 5:**
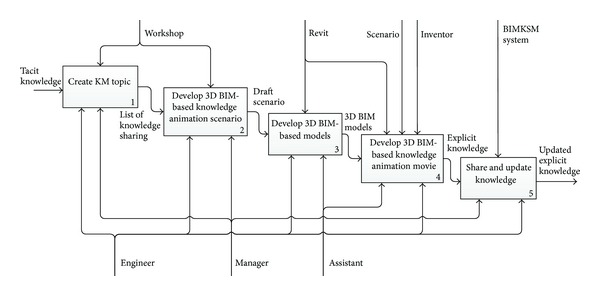
The BIM-based knowledge animation usage process.

**Figure 6:**
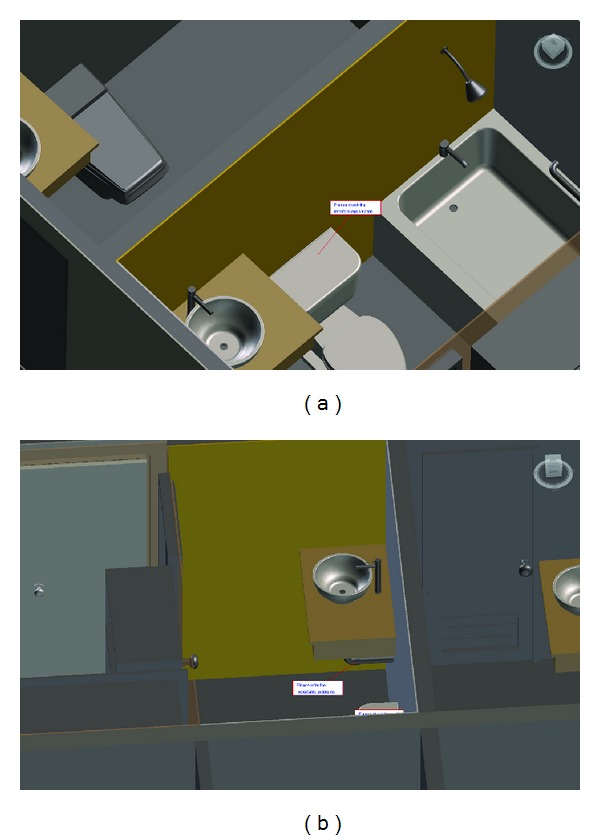
Application of BIM-based knowledge models for knowledge sharing in the case study.

In the following case, the architecture firm has had sixteen years of experience specifically in construction building projects. The architecture firm hoped to take full advantage of the BIM-based knowledge management system to facilitate knowledge exchange and management during the construction phase and reuse it in other similar projects. Therefore, the architecture firm announced that all jobsite and project managers would be encouraged to use the BIMKSM system to apply knowledge management in order to effectively manage acquired information during the construction phase in the BIM environment. The BIMKSM system was utilized in the construction project to verify the proposed methodology and demonstrate the effectiveness of sharing previous experience in the construction phase. The project used as a case study lasted 4 months. During the study, all jobsite and project managers were encouraged to explore and edit their own recorded experiences in the BIMKSM system.  Figure 5 shows the BIM-based knowledge animation usage process.  Figure 6 illustrates application of BIM-based knowledge models for knowledge sharing in the case study.

As previously mentioned, the case study was implemented in the middle of the construction phase. All BIM models used in the study were created and developed for the purpose of construction management. Finally, the BIM models were reused and applied for knowledge management.

First, the engineers were invited to explain their experiences and provide comments based on the issue and include relevant information and documents. The initial engineer created issues regarding the selected activity and objects of the BIM model in the initial phase. All related documents for this issue were collected and digitized by the senior engineers and knowledge assistants. After the issue was created, the senior engineers were invited to share their knowledge and comments related to the issue using the system. The posted files included digital documents, photos, and films. The knowledge assistants helped the senior engineers to digitize the content, and they then created the BIM objects related to the issue. The other issues were communicated in the same manner. All engineers were required to submit experience-based information and discussions regarding the issue via the BIMKSM system. The engineers read previous comments provided by others, learned from these records, and submitted their own comments via the BIMKSM system, which then allowed other engineers to discuss their work. The comments provided by the senior engineers included notes, actual problems/solutions, and suggestions. The engineers communicated problems and solutions to the senior engineers, posted their comments in the system, and shared their case discussions with others. The engineers were required to submit their knowledge pertaining to the BIM objects of the BIM model via the BIMKSM system. The senior engineers reviewed all questions and solutions and posted comments for all interested individuals. Furthermore, all information was stored in the central database to prevent the collection of redundant data. Finally, the information was automatically backed up from the system database to another database. Moreover, the knowledge was updated later because further feedback and another solution to the same problem were provided. The updated knowledge set was republished in the object of BIM model of the BIMKSM system after the approval process was completed. A notice message was then transmitted to authorized members.

### 5.2. Case Study Evaluation

Questionnaire results from the case study evaluation reveal that the BIMKSM system effectively shares knowledge. A verification test was performed by checking whether the BIMKSM system could perform tasks as specified in the system analysis and design. A validation test was undertaken by requesting selected case project participants to use the system and then providing feedback by answering a questionnaire. There were 18 respondents: two project managers with ten years of experience; six jobsite engineers with ten years of experience; seven jobsite engineers with five years of experience; two assistants with three years of experience; and one CKO with ten years of experience. The BIMKSM system was demonstrated to the respondents, who were requested to give their opinions on it by completing the questionnaire.  Table 1 shows the results of the system evaluation.

Overall, the jobsite engineers' feedback for the case study was positive. Most engineers and project managers agreed that the BIMKSM system helps them to view all collected knowledge and experience in the BIM environment. Questionnaire results indicate that the primary advantages of using the BIMKSM system are as follows: (1) it provides 3D visual illustration of knowledge regarding project-based knowledge (86% agreed); (2) it provides BIM-assisted animation easily and effectively (89% agreed); and (3) it clearly identifies available knowledge and different status of different knowledge in the BIM environment (90% agreed).

Questionnaire results indicate that the primary advantages of the application of BIM in BIM-based knowledge models are as follows: (1) it provides clear 3D representations, thus identifying knowledge and experience feedback relevant to an object or activity (92% agreed); (2) one can generate a visual object of BIM model illustrations of knowledge, thus identifying acquired knowledge and experience feedback relevant to tasks and projects (90% agreed); (3) it allows one to view knowledge and information provided by jobsite engineers easily and effectively (90% agreed); and (4) it enables engineers to trace and manage any acquired BIM-based knowledge feedback (88% agreed).

User feedback indicated that the primary barriers to using the BIMKSM system were as follows: (1) insufficient updated information is related to different types of knowledge; (2) substantial amounts of time and assistance are needed for engineers and managers to use BIM software to edit and update knowledge feedback; (3) further effort is required to update information related to various objects of a BIM model or the activities in a project; (4) the senior and jobsite engineers require substantial time and assistance to edit knowledge feedback in BIM environment; and (5) few engineers do not accept BIM applications in the construction sites.

## 6. Conclusion

To improve construction knowledge sharing in building projects, this work presented and developed the BIMKSM system as a visual platform. The BIMKSM system illustrates knowledge with problem descriptions and solutions in the BIM environment. BIM is a highly promising means of enhancing knowledge management during the construction phase of a project. Collecting problem descriptions and solutions using the BIM technology allows project managers and jobsite engineers to contribute and share the most up-to-date knowledge and experience regarding problems and solutions in construction. The BIM technology generates 3D drawings, thus identifying valued experience-based knowledge relevant to issues and activities. Additionally, BIM provides objects and illustrations when knowledge is available. The BIMKSM system collects specific problem solutions and supports all information across projects. Overall, field test results indicate that the BIMKSM system is an effective and simple platform for knowledge management in construction projects. The case study results demonstrate the effectiveness of a BIMKSM-like system for KM due to its incorporation of BIM and web technologies during the construction phase.

The concept of a BIM-based knowledge management system was presented, as was a system for use as a knowledge-sharing platform in construction design-build projects. The application of BIM-based knowledge management system mainly allows project participants to access the knowledge easily and effectively. Although effort is required to update information on various problems and solutions, the proposed system benefits KM by (1) developing web-based BIM-assisted knowledge management system for construction knowledge sharing, (2) providing an effective and efficient method to assist and manage visual KM work, (3) enabling users to learn knowledge through BIM-based knowledge animation.

In sum, the engineers were able to increase their understanding of previous captured knowledge and experience from all participants working on different projects. Notably, BIM integrates the objects comprising knowledge management work by incorporating external factors, such as problem and solution descriptions, comments, and suggestions, into a single source for all construction BIM-based KM information. Effectively utilizing web technologies and BIM during the construction phase allows project participants to enhance knowledge sharing for domain knowledge management. The case studies also show that a BIMKSM system can provide a web-based visual BIM-assisted knowledge sharing management platform.

## Figures and Tables

**Table 1:** System evaluation results.

System evaluation item	Mean score
Enhances visual knowledge illustration	4.5
Applicability to construction knowledge management	4.8
Reduces rework percentage	4.2
Reduces percentage of mistakes occurring	4.3
Improves the knowledge collection	4.2
Enhances knowledge communication	4.4
Improves knowledge sharing	4.7
Enhances learning performance	4.3

The mean score is calculated from respondents' feedback on a five-scale questionnaire: 1 (strongly disagree), 2, 3, 4, and 5 (strongly agree).
